# Ultrasound screening protocol for osteochondrosis at selected predilection sites in thoroughbred yearlings

**DOI:** 10.1186/s13620-022-00216-7

**Published:** 2022-04-27

**Authors:** Seamus Hoey, Jeremiah O’Sullivan, Jennifer Byrne, Sinead Devine, William Toomey, Hester McAllister, Cliona Skelly

**Affiliations:** 1 Equine Clinical Studies, Diagnostic Imaging and Anaesthesia, School of Veterinary Medicine, University College Dublin, Dublin, Ireland; 2 Clinic na gCapall, Farranacurragh, Oldleighin, Co. Carlow Ireland

**Keywords:** Equine, Joint, Osteochondrosis, Screening, Ultrasound

## Abstract

**Background:**

Osteochondrosis is a common condition of young horses where there is a failure of endochondral ossification, usually at predisposed sites. The estimated prevalence of osteochondrosis is 33–44%, with radiographic screening of yearlings being used to identify lesions. Radiography has two major limitations: poor sensitivity in detecting cartilaginous lesions and secondly, the exposure of the horse and personnel to ionising radiation. Ultrasonography allows imaging of the articular cartilage and subchondral bone margins and has been shown to be more sensitive in identifying osteochondrosis lesions. However, the ultrasonographic technique for examining joints is operator dependant, resulting in highly variable examinations, thus affecting its reliability and reproducibility as a screening test.

**Results:**

A prospective observational clinical population study was undertaken involving twenty-two clinically normal weanling thoroughbred horses on-farm, describing a detailed protocol of the ultrasonographic examination technique for on-farm screening of common sites of osteochondral disease in the young horse, namely the carpal, metacarpophalangeal, stifle, tarsal and metatarsophalangeal joints.

**Conclusion:**

Two veterinary practitioners used the technique to illustrate the repeatability of the protocol. The step-by-step protocol provides a valuable, reliable, repeatable technique for veterinary professionals performing screening ultrasound in the field.

**Supplementary Information:**

The online version contains supplementary material available at 10.1186/s13620-022-00216-7.

## Background

Osteochondrosis is the most important developmental orthopaedic disease in horses [1] characterised by failure of endochondral ossification in the epiphyseal or metaphyseal growth plates, resulting in an area of thickened cartilage [2]. These areas may ossify and return to an almost normal condition. Alternatively, cartilage necrosis may develop resulting in a fissure in the cartilage or subchondral bone, with subsequent cartilage flap formation, development of subchondral osseous cyst like lesions or collapse of the articular cartilage [3, 4]. With a shear stress, a cartilage flap fragment can develop. This fragment can detach leading to a free intraarticular body or “joint mouse”, corresponding to osteochondrosis dissecans [5]. Osteochondrosis has a prevalence of 33–44% in horses, most frequently seen in Thoroughbreds and Warmblood horses [6–8]. Lesions may develop within the first seven months of age, but can be clinically silent until the horse is brought into work [9, 10]. In the weanling and yearling horses the most commonly affected joints are the tarsocrural, femoropatellar, metacarpophalangeal and metatarsophalangeal joints, followed by the carpal, elbow, shoulder and cervical articular facet joints [11, 12].

A standardised set of screening “yearling radiographs” are acquired to highlight any abnormality which may affect the future racing and breeding potential [13]. Osteochondrosis lesions can negatively impact the price at sale [14, 15]. As part of the routine sales process each year, countless horses are exposed to ionising x-ray radiation, and the teams of personnel acquiring the images are also exposed to scatter radiation [16].

Equine joint ultrasound examinations are usually focused in nature where lameness is localised to a joint or set of joints. The common use of ultrasound in general practice facilitates the majority of equine practitioners have access to ultrasound equipment, with good quality, reasonably priced, mobile ultrasound machines [17].

Ultrasonographically, articular cartilage is a smooth hypoechoic to anechoic band, superficial to a smooth curvilinear hyperechoic surface eliciting distal acoustic shadowing, consistent with the cartilage-subchondral bone interface [18]. Several authors describe the evaluation of the periarticular and articular anatomy of the joints, as well as specific examples of disease [2, 18–32]. Osteochondrosis can manifest as an interruption in the subchondral osseous margin, depressions or semi-circular indentations in the cartilaginous or subchondral interface [17, 22]. However, ultrasound technique is very operator dependant and interpretation of the images acquired may be difficult when assessed by a reviewer other than the ultrasonographer.

The aim of this study is to develop a step- by step ultrasound protocol for on-farm screening of horses for the most common sites of osteochondrosis that can be performed by veterinary practitioners and produce standardised images.

## Results

Twenty Two weanling Thoroughbred horses with a mean age of 314 days (range of 281–367 days) were included in the study, 11 male and 11 female. Ultrasonographic studies were performed in less than one hour (15 to 58 min). With practice, the time frame for completing the ultrasound examination was between 15 and 35 min.

Administration of ample amounts of warmed alcohol facilitated good image quality despite the unclipped hair. The articular cartilage and subchondral bone margin were readily visualised. Frequently a thin hyperechoic line was identified superficial to the hypoechoic cartilage, representing the cartilage-synovial fluid interface.

### Carpal joints

**Fig. 1:**
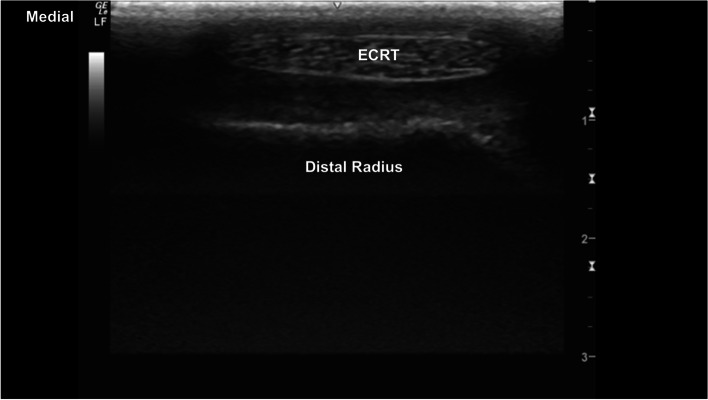
Transverse image at the craniodistal antebrachium, centred at the extensor carpi radialis, superficial to the hyperechoic distal radial epiphysis. The extensor carpi radialis is identified superficially with well defined margins. The distal radial epiphysis is deep to the extensor carpi radialis with mildly irregular hyperechoic margins and distal acoustic shadowing. *Marker is to medial*

**Fig. 2:**
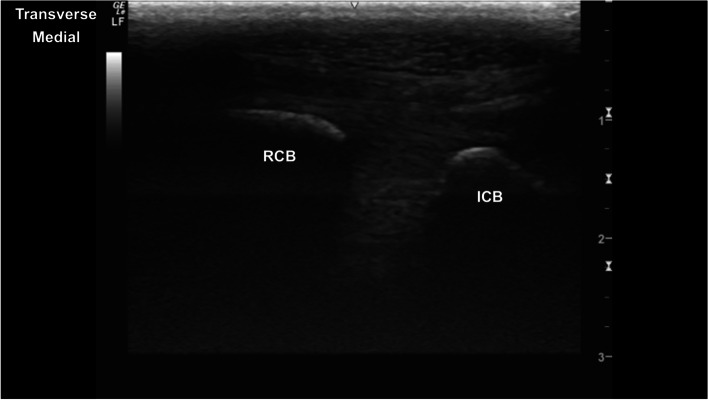
Transverse image of the dorsal carpus at the interface between the smoothly marginated hyperechoic radial and intermediate carpal bones. *Marker is to medial*

**Fig. 3:**
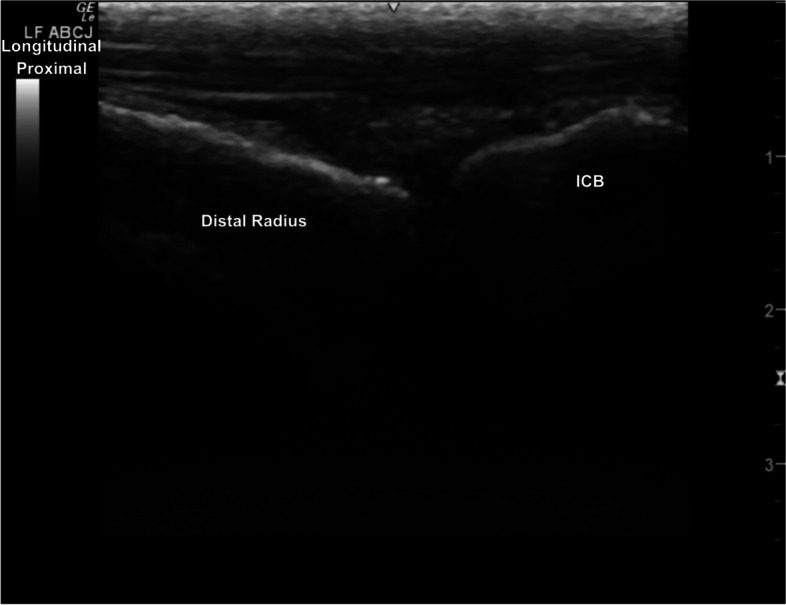
Longitudinal image of the dorsal carpus, with well-defined hyperechoic margins of the left distal radius and the intermediate carpal bone and the hypoechoic antebrachiocarpal joint space. *Marker is to proximal*

**Fig. 4:**
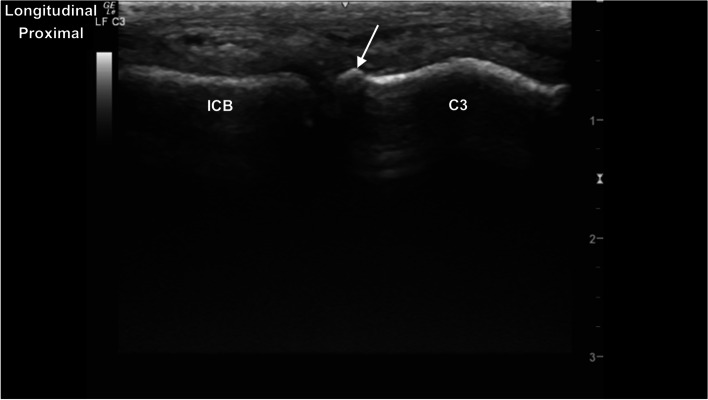
Longitudinal image of the dorsal carpus of the middle carpal joint, with hyperechoic extension at the dorsoproximal aspect of C3 (arrow), with surrounding mild hypoechoic material and minimal narrowing of the middle carpal joint space. *Marker is to proximal*

**Fig. 5:**
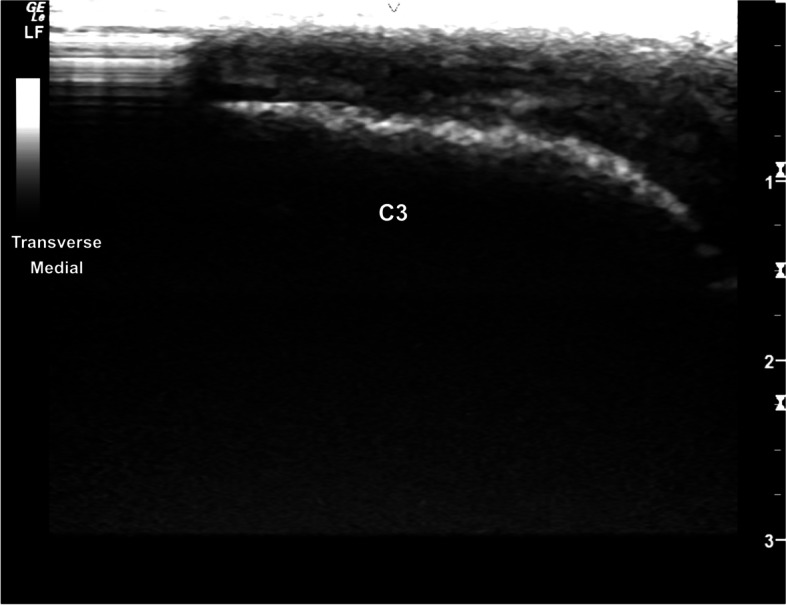
Transverse image of the dorsal carpus, showing a mildly undulant dorsoproximal margin of C3. *Marker is to medial*

The extensor carpi radialis was easily identified at the mid-cranial aspect of the distal radial physis (Fig. 1). The hyperechoic margins of the carpal bones were smooth and well defined. The intercarpal joint spaces were easily visualised with well-defined intercarpal ligaments (Fig. 2). The antebrachiocarpal joint (ABCJ), middle carpal joint (MCJ) and carpometacarpal (CMCJ) margins are sharp, with narrow hypoechoic interfaces. The adjacent radial, carpal, and metacarpal bones were well defined (Fig. 3). Mild smooth hyperechoic projections from the articular margins of the carpal bones were identified, with distal acoustic shadowing and normal adjacent articular margins of the opposing bone (Fig. 4). Minimal irregular undulation of the hyperechoic osseous margins was seen at the dorsoproximal extent of the carpal bones on transverse images, with the third carpal bone (C3) most affected (Fig. 5).

### Metacarpophalangeal/metatarsophalangeal joints

**Fig. 6:**
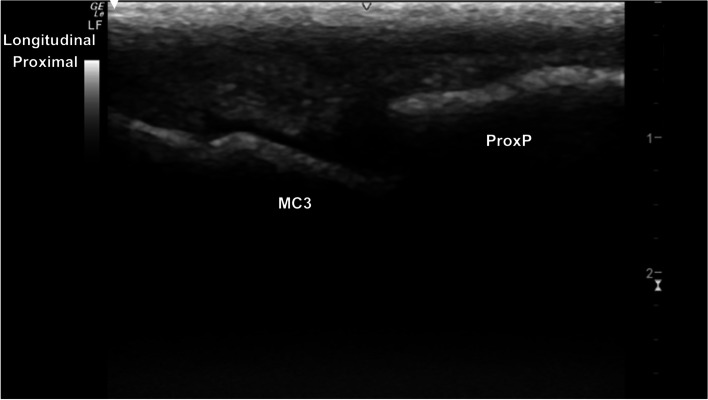
Longitudinal mid-sagittal dorsal image of the metacarpophalangeal joint. The third metacarpal bone is to the left and the proximal phalanx to the right of the image. A thin well-defined hypoechoic region superficial to the hyperechoic subchondral bone represents the normal cartilage of the distal condyle of the third metacarpal bone (MC3) (arrow). *Marker is to proximal*

**Fig. 7:**
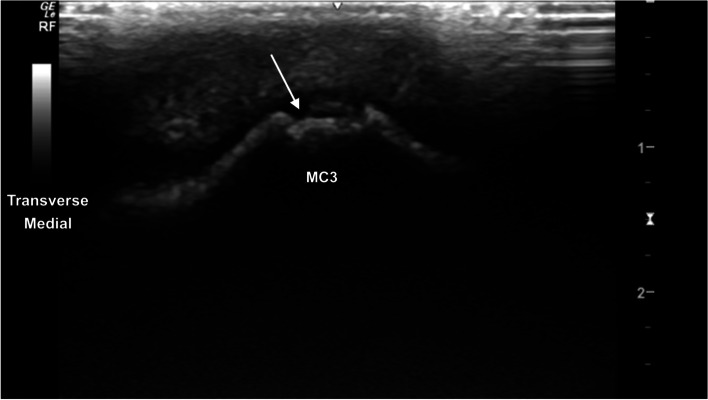
Transverse mid-sagittal dorsal image of the sagittal ridge of the third metacarpal bone. A thin mid-sagittal irregularly marginated hypoechoic defect is seen at the sagittal ridge (arrow), with irregular subchondral hyperechoic margins and heterogeneous hyperechoic material within the defect. Mild undulant thickening of the superficial articular cartilaginous margins *Marker is to medial*

**Fig. 8:**
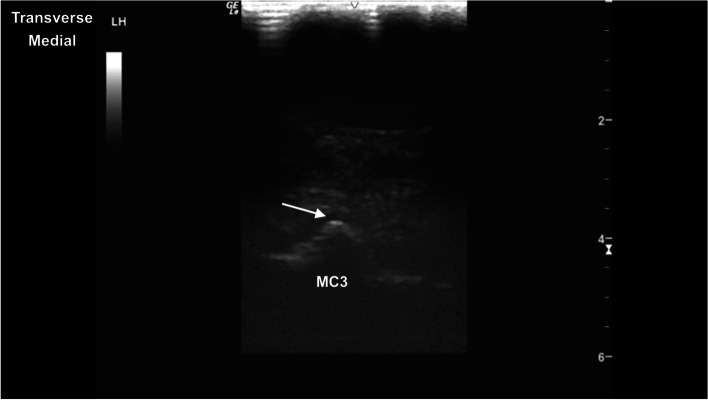
Transverse mid-sagittal plantar image of the sagittal ridge of the third metatarsal bone. The distal sesamoidean ligaments are superficial to the third metatarsal bone. The sagittal ridge is identified with a thin hypoechoic cartilaginous rim (arrow) and deeper hyperechoic subchondral bone, and distal acoustic shadowing. *Marker is to medial*

**Fig. 9:**
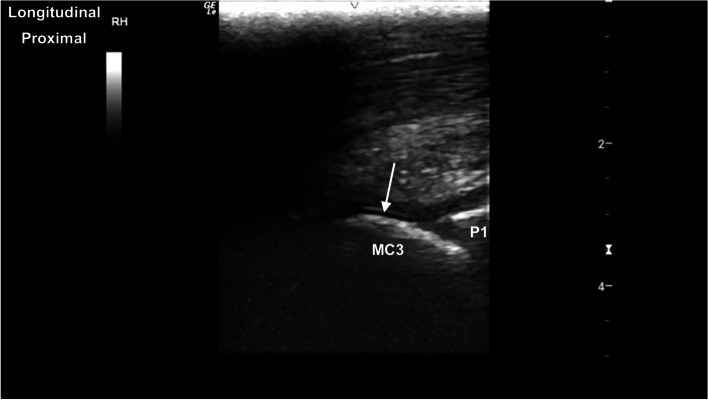
Longitudinal mid-sagittal palmar view of the distal third metacarpal bone. The thin curvilinear hypoechoic cartilage (arrow) and deeper hyperechoic subchondral bone of the sagittal ridge is to the left, with the proximal aspect of the proximal phalanx (P1) to the right. *Marker is to proximal*

**Fig. 10:**
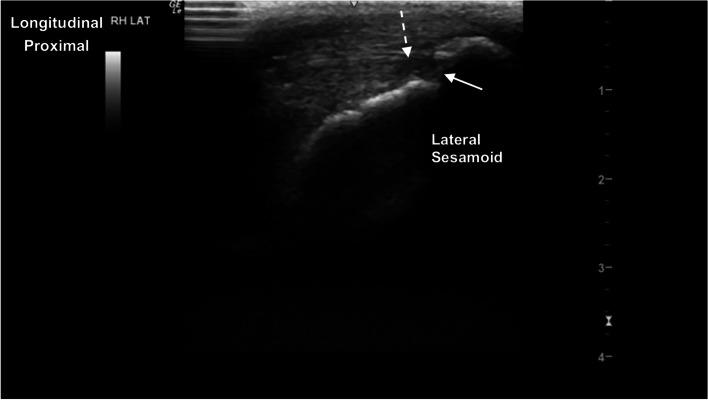
Longitudinal parasagittal image of the right hind lateral sesamoid. Moderate undulation of the proximal margin of the sesamoid bone, and a well-defined hypoechoic defect (arrow) at the plantar margin. There is corresponding hypoechoic material at the insertion of the lateral branch of the tendon of the third interosseous medius muscle (suspensory ligament) (dashed arrow). *Marker is to proximal*

**Fig. 11:**
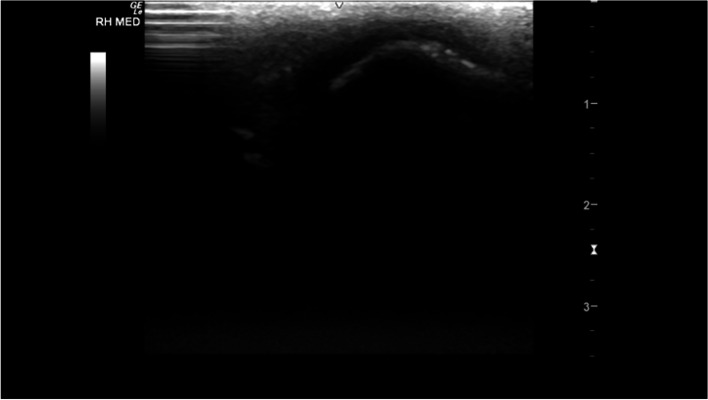
Longitudinal parasagittal plantar image of the well-defined hyperechoic margin of the right hind medial plantar eminence of the proximal phalanx, immediately distal to the medial sesamoid bone. *Marker is to proximal*

The metacarpophalangeal (MCPJ) and metatarsophalangeal joints (MTPJ) were the most technically challenging joints to evaluate, in both standing and partial flexion. Standing, the dorsal margin of the sagittal ridge of the distal third metacarpal/metatarsal bone (MC3/MT3) showed well-defined cartilage and subchondral bone margins (Fig. 6). The dorsodistal margin of the sagittal ridge frequently showed irregular subchondral bone margination, with hypoechoic linear to irregular echogenicities (compared to the subchondral bone margin) deep to the normal articular cartilage margins (Fig. 7). In partial flexion the sagittal ridge was more ill-defined when imaged through the intersesamoidean ligament (Figs. 8 and 9). Mild undulation and irregularity of the osseous margins of the sesamoid bones was frequently identified. Mild indentations of the hyperechoic interface were seen with and without focal areas of hypoechogenicity of the adjacent attachment of the branches of the tendon of the third interosseous medius muscle (previously referred to as the suspensory ligament) (Fig. 10). The palmaroproximal aspects of the palmar eminences of the proximal phalanx (P_1_) were imaged by minimal fanning of the transducer in a palmaroproximal-dorsodistal direction (Fig. 11).

### Stifle joint

**Fig. 12:**
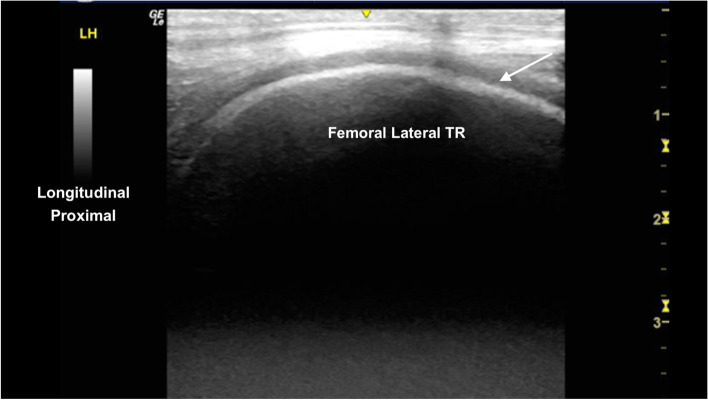
Longitudinal craniolateral image of the lateral trochlear ridge (TR) of the femur, showing a smooth curvilinear hyperechoic subchondral bone margin and uniform superficial hypoechoic cartilage (arrow). *Marker is to proximal*

**Fig. 13:**
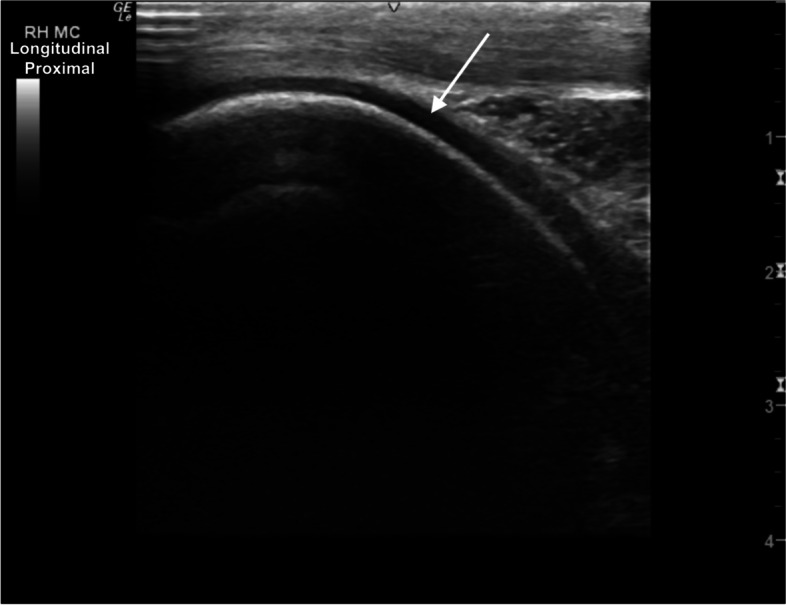
Longitudinal craniomedial image of the medial femoral trochlear ridge, showing a smooth hyperechoic subchondral bone margin and uniform superficial hypoechoic cartilage (arrow). *Marker is to proximal*

**Fig. 14:**
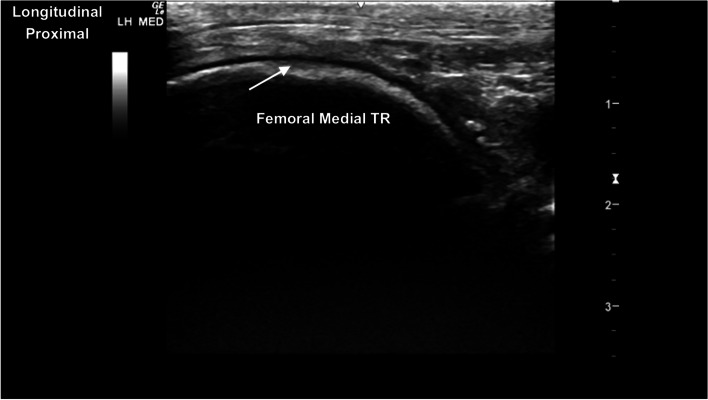
Longitudinal craniomedial image of the medial femoral trochlear ridge. There is a region of mild blunting and thickening of the hyperechoic subchondral bone margin (arrow) and minimal irregular thickness of the hypoechoic superficial cartilage. *Marker is to proximal*

**Fig. 15:**
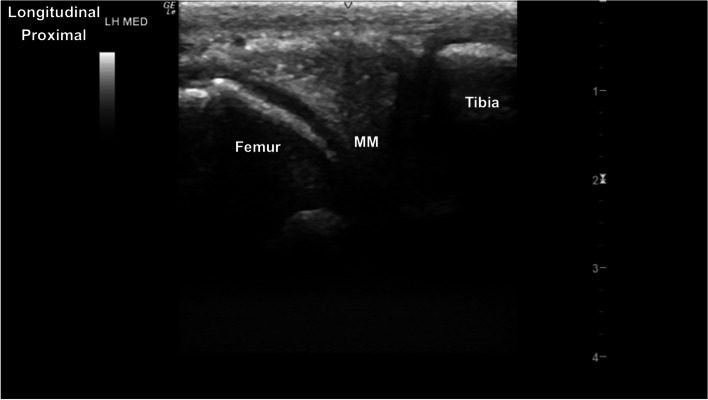
Longitudinal image of the medial aspect of the stifle joint. The triangular heterogeneously hyperechoic medial meniscus (MM) is between the medial femoral condyle and medial aspect of the tibial condyle. The curvilinear hypoechoic medial femoral condylar cartilage and deeper hyperechoic subchondral bone are identified, and the medial margin of the left tibial condyle. *Marker is to proximal*

The femoral trochlear ridges (TRs) were well defined and smoothly marginated showing hyperechoic subchondral bone and hypoechoic superficial cartilage (Figs. 12 and 13). There was mild blunting of the craniodistal subchondral bone margin of the medial femoral TR, with equivocal heterogeneity of the superficial cartilage layer (Fig. 14). The femoral and tibial condyles are well defined, surrounding the menisci (Fig. 15).

### Tarsal joints

**Fig. 16:**
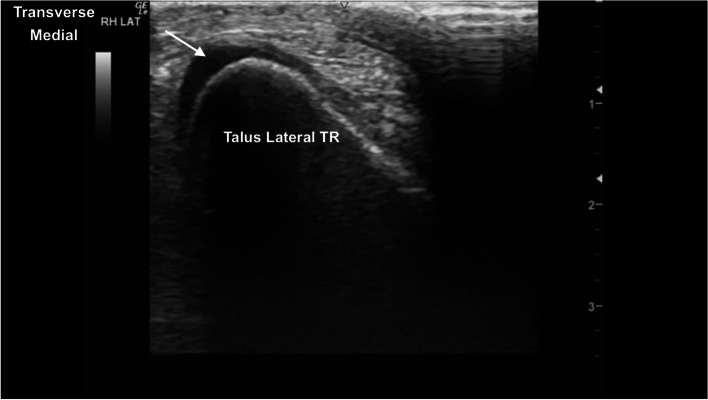
Transverse dorsolateral image of the lateral trochlear ridge of the talus. The hyperechoic subchondral bone is curvilinear and well defined, with superficial hypoechoic cartilage. There is normal mild thickening of the dorsoaxial hypoechoic cartilage (arrow). *Marker is to medial*

**Fig. 17:**
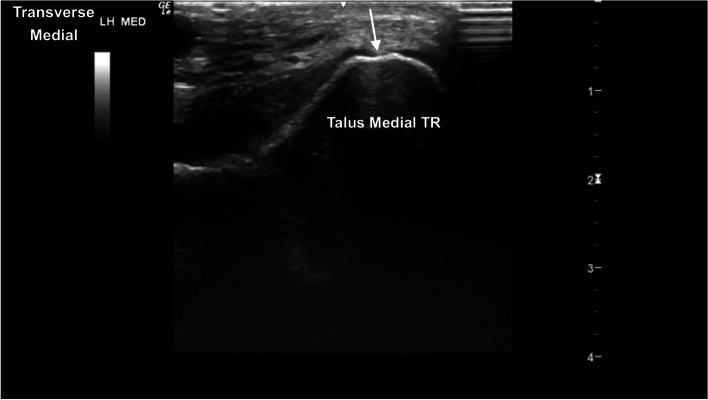
Transverse dorsomedial image of the medial trochlear ridge of the talus. Mild indentation of the curved hyperechoic subchondral bone, with mild thinning and hyperechogenicity of the superficial cartilage (arrow), seen frequently. *Marker is to medial*

**Fig. 18:**
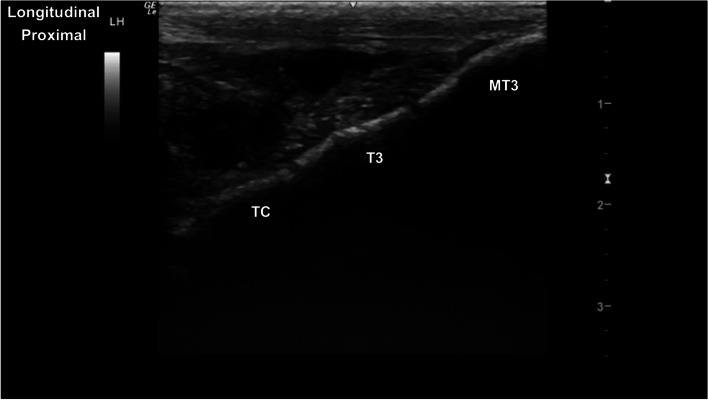
Longitudinal parasagittal dorsomedial image of the normal sharply delineated hyperechoic central tarsal bone (TC), third tarsal bone (T3) and the third metatarsal bone (MT3) margins. Normal centrodistal and tarsometatarsal joint spaces. *Marker is to proximal*

**Fig. 19:**
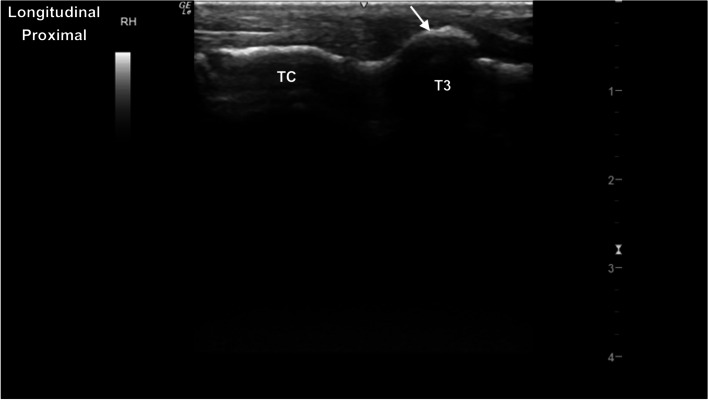
Longitudinal parasagittal dorsal image of the tarsus, centred at the centrodistal tarsal joint. Note the mild smoothly demarcated dorsal hyperechoic protuberance of the third tarsal bone (T3). *Marker is to proximal*

**Fig. 20:**
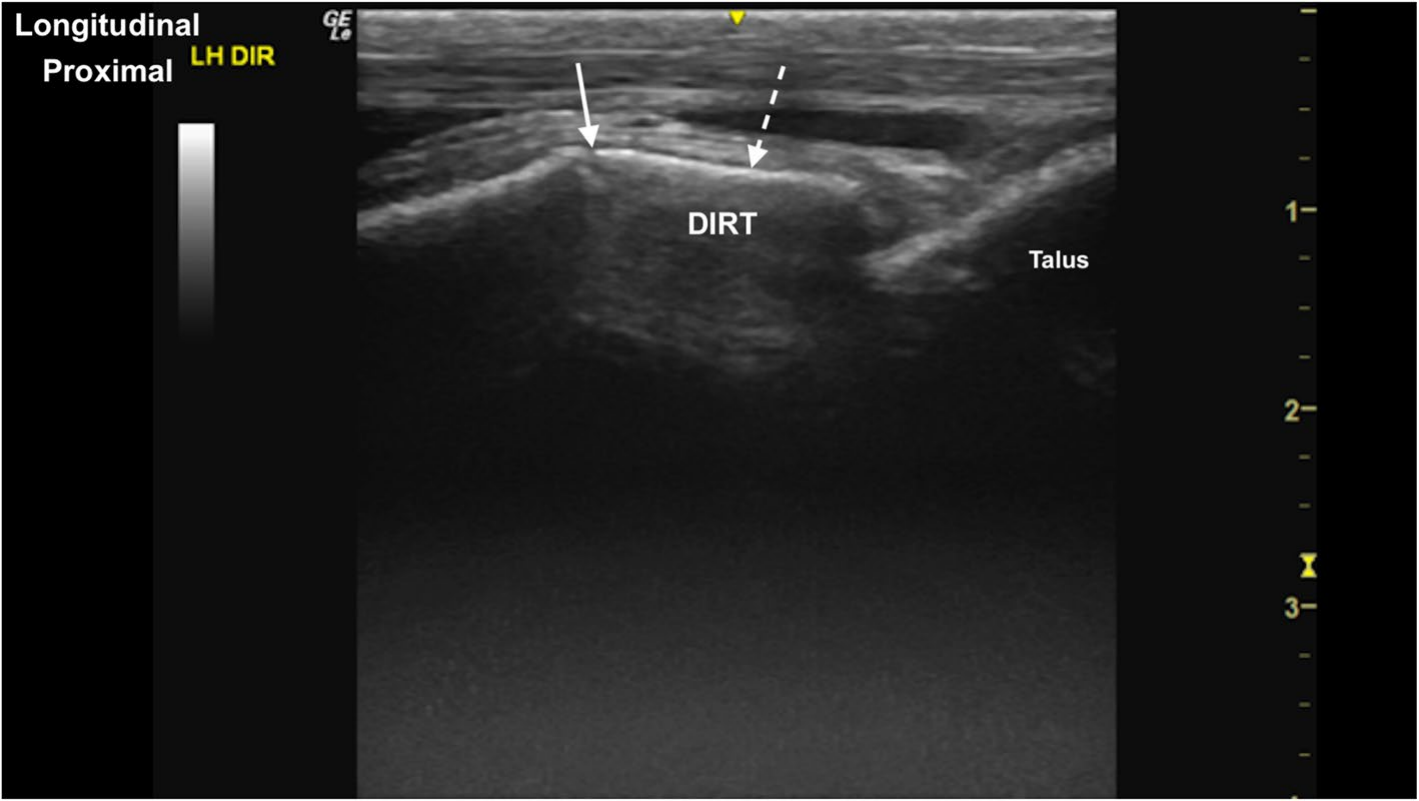
Longitudinal parasagittal cranial image of the distal intermediate ridge of the tibia (DIRT). The distal intermediate ridge appears as a linear (dashed arrow) hyperechoic structure. There is a normal well-defined hypoechoic interface (arrow) with the remainder of the distal tibial epiphysis. *Marker is to proximal*

**Fig. 21:**
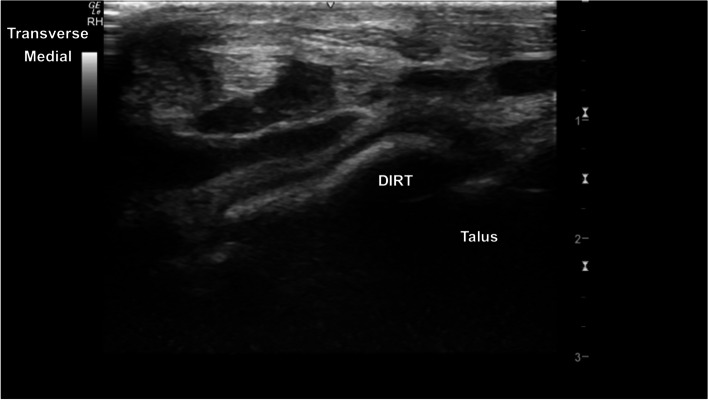
Transverse parasaggital cranial image of the distal intermediate ridge of the tibia. The distal intermediate ridge is superficial to the medial and lateral trochlear ridges of the talus and the trochlear groove. *Marker is to medial*

**Fig. 22:**
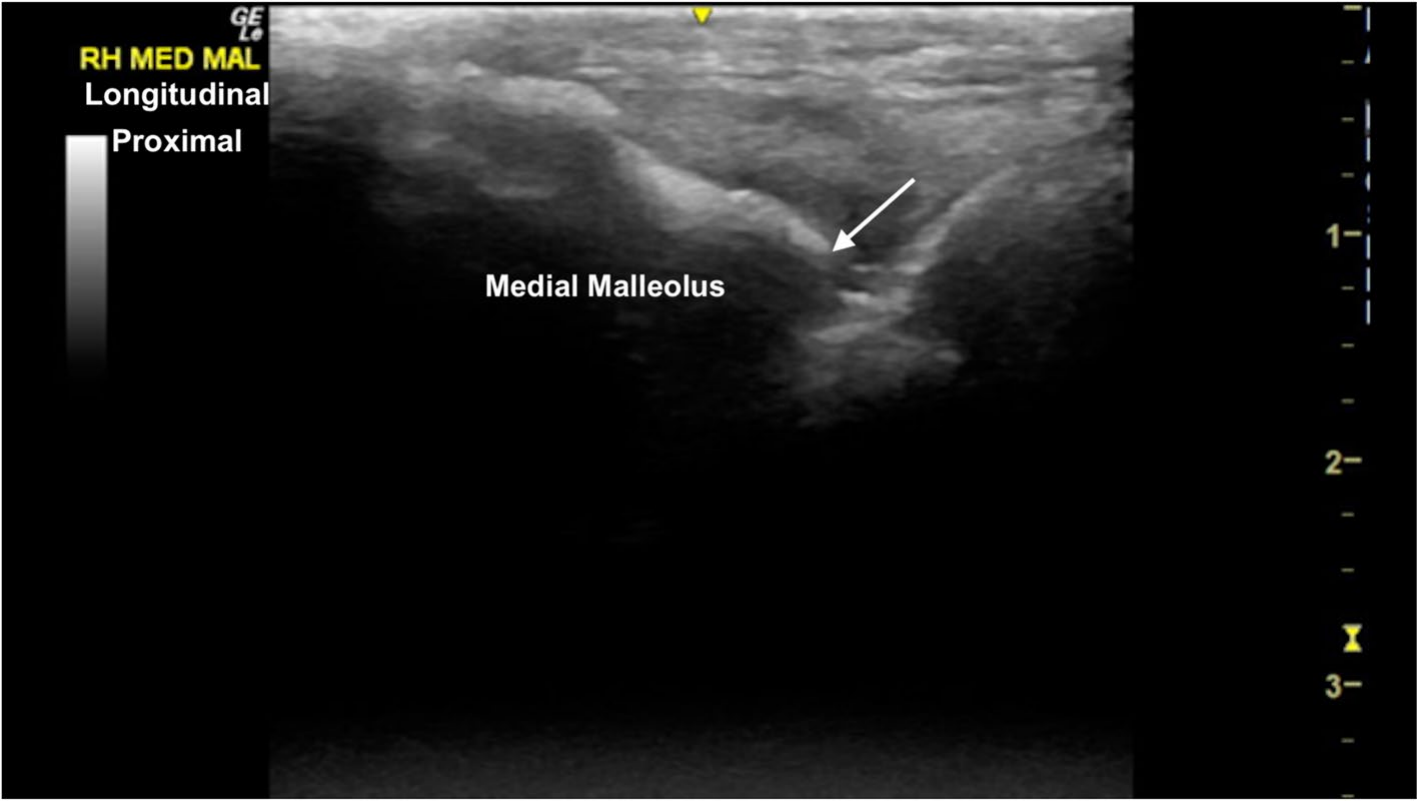
Mildly oblique longitudinal image of the medial aspect of the tarsocrural joint, centred on the left medial malleolus. The medial malleolus is hyperechoic with rounded margins distally (arrow), extending axially at the level of the tarsocrurual joint. *Proximal is to the left*

The TRs of the talus showed well-defined smoothly curved subchondral bone margins (Fig. 16). Mild indentation of the subchondral bone and thinning of the superficial cartilage was identified intermittently at the cranioproximal aspect of the medial femoral TR, without any evidence of joint effusion or irregular margins, likely a normal variant (Fig. 17). The central and distal rows of tarsal bones showed well-defined articular margins (Fig. 18). The well-defined dorsal protuberance of the third tarsal bone showed a smooth extension of the hyperechoic osseous margin (Fig. 19). The distal intermediate ridge of the tibia (DIRT) was clearly visible (Fig. 20), with mild proximomedial to distolateral rotation necessary due to the oblique orientation of the TRs of the talus (Fig. 21). The longitudinal view of the medial malleolus required fine proximomedial to distolateral rotation and rocking of the transducer. There was some undulation of the medial margin of the medial malleolus in several horses (Fig. 22).

### Screening protocol pilot

One veterinary practitioner; sonographer 2, had 20 years of clinical experience and felt moderately experienced in ultrasound and mildly experienced in equine joint ultrasound. Sonographer 2 reported that the technique was relatively easy to perform and spent 40 min and 22 s on the first examination without stifle flexion, and 38 min and 58 s on the second examination with stifle flexion.

The second practitioner; sonographer 3, had 2.5 years of experience and reported being relatively inexperienced in ultrasound. Sonographer 3 reported that the technique was somewhat difficult to perform and spent 45 min and 22 s on the first examination without stifle flexion, and 39 min and 49 s on the second examination with stifle flexion.

On specific joints, both reported that images of the carpus, metacarpophalangeal (in flexion and extension), metatarsophalangeal (in flexion and extension) and stifle (in flexion and extension) joints were relatively easy to very easy to acquire. Both observers reported that the tarsus was relatively difficult, with both reporting that the speed and difficulty of image acquisition improved with practice.

## Discussion

A systematic protocol of the ultrasonographic assessment of a selection of the most common sites of osteochondrosis in yearling horses is described in detail, facilitating the use of the protocol by veterinary practitioners with variable experience.

Radiography is currently the recognised gold standard for equine orthopaedic screening, particularly in the case of pre purchase examinations. A major limitation of radiography is the inability to distinguish cartilage from the other soft tissue opacities of the joint. In radiography, the deformation of the subchondral bone margins, change in opacity of the subchondral bone and soft tissue swelling are signs suggestive of osteochondral disease. Subtle cases involving only the articular cartilage may be missed.

Ultrasound can directly image cartilage and the subchondral bone margin, and without exposure to ionising radiation. Ultrasonographic detection of osteochondrosis of the canine shoulder and the equine tarsocrural joints have been described [33], and show a higher sensitivity in the detection of osteochondrosis, compared to radiography [23, 34]. In humans, ultrasound screening for osteochondrosis lesions in young athletes describe positive predictive values of 66.7 to 100%, when compared to MRI. Indeed, the measurement of defects identified ultrasonographically were similar to those of MRI [35, 36].

All ultrasound examinations of the weanling thoroughbred horses performed in early spring. The haircoat at this time was not very long or dense, and the fine hair and skin of the thoroughbred weanling permitted adequate ultrasound beam penetration and quality image acquisition. In some cases, the area of interest was brushed to remove dirt or debris from the hair and to allow direct alcohol application to the skin and hair. In some cases, repeat application of alcohol was necessary for optimal imaging, which was well tolerated by the horse. It was not possible to clip the joints of these horses for the examinations, and in clinical practice it would not be feasible to clip each horse for ultrasound on aesthetic grounds. Ultimately all joints in all horses were imaged fully with good image quality yielding comparable, repeatable images.

Despite the ubiquitous presence of ultrasound in general equine practice, there is little literature describing a standardised technique in joint imaging. Many texts describe the normal ultrasonographic anatomy of joints, with an emphasis on the soft tissue structures such as periarticular tendons, ligaments, muscles, tendon sheaths and bursae as well as the joint capsule and synovium. The specific ultrasound technique is rarely or incompletely described [2, 20, 25–31, 37, 38]. The inconsistency in ultrasound technique results in images that are often not comparable with follow up images, or that the images can’t be reliably assessed by anyone other than the sonographer. In radiography there is a defined collection of radiographic views acquired for optimal assessment of the yearling horse at the time of sale. These views are commonly performed in specialty and first opinion practice and are thus repeatable and comparable tests. In ultrasound examinations, the skill of the ultrasonographer in the handling and manipulation of the ultrasound transducer can vary greatly. Images recorded in an ultrasound examination can be variable in the anatomy imaged, the imaging plane/transducer orientation and the ultrasound imaging settings (such as focal zone placement, frequency, transducer used, gain settings). This limits the repeatability and comparability of the images between horses, and between ultrasonographers. By describing a detailed protocol indicating optimal transducer orientation and movements, the imaging planes can be standardised facilitating repeatable and comparable image recording. In this way, recorded images can be subsequently reviewed and compared between horses and between time points.

The initial decision to remove the flexed stifle for the additional observers was the perception that imaging the flexed stifle in a yearling would be difficult and time consuming. The observers did not perceive the flexed stifle as a more difficult component of the examination, and the visualisation of the more caudodistal aspects of the femoral condyles would be important in the search for osteochondral disease. Both observers noted that imaging the tarsus was more challenging reflecting the more complex anatomy. However, confidence improved with practice, and both observers acquired diagnostic images. Both practioners highlighted the importance of having reference images to emulate ensuring the images acquired were consistent and repeatable.

A limitation of ultrasound is that the ultrasound waves are unable to penetrate bone, and as a result restrict ultrasonographic assessment of deeper structures. In this study movement and flexion of the stifle and metacarpophalangeal joints were used to better interrogate deeper articular margins. Despite this limitation, most common sites of osteochondrosis of the fore and hindlimb joints can be thoroughly accessed over the course of the examination. Diagnostic images could be acquired through the use of warmed alcohol as a couplant placed directly on haired skin. By removing the need to clip these weanlings, the screening process is more attractive to owners and trainers.

The images acquired of the clinically normal weanling horses showed multiple repeatable echogenic changes within the trochlear ridges of the femur and talus, and within the sagittal ridge of the metacarpal and metatarsal bones. These changes were not associated with joint effusion or swelling. Futher study would be required to compare the ultrasonographic findings to the radiographic findings at the time of ultrasound, and to the radiographic findings at the time of yearling radiographic imaging.

The technique does not include all the sites of disease in the juvenile horse but focuses on the most common sites of osteochondral disease. By moving through the imaging planes, cineloops can be recorded, documenting the most common sites of osteochondral disease. The imaging planes would include the osteochondral sites of osteochondrosis as well as the dorsal aspects of the joints, where osteochondral (OCD) fragments may be identified. The site of origin of osteochondrosis may be identified, where identification of free osteochondral fragments may not be captured in the imaging planes, a diagnosis of osteochondrosis would remain. These cineloop images can be reviewed by the ultrasonographer as well as another observer, where images are repeatable and comparable. Further studies are warranted, comparing the ultrasound protocol with contemporaneous radiographic examinations. The ultrasound examination protocol may be useful as an initial screening test, where any equivocal findings could be further examined using radiography. The screening protocol would not replace a diagnostic examination targeted at a specific site of lameness or pain.

## Conclusion

An ultrasonographic screening protocol is described in a detailed systematic manner facilitating on-farm screening using mobile ultrasound equipment. The protocol has been shown to be feasible for practitioners of varied experience in ultrasound, thus can have a role as a potential screening method capable of reproducing standardised images, in a reasonable time frame with only minimal practice.

## Materials and methods

Thoroughbred weanlings were included as part of the annual screening programme on three farms. A clinical examination of each weanling demonstrated normal gait at walk and normal cardiac auscultation. Light sedation using intravenous administration of 3.5 mg of Detomidine (Domosedan, Zoetis) 3.5 mg of Butorphanol (Torbugesic, Zoetis) and 3.75–5 mg of Acepromazine (Calmivet, Vetoquinol).

The ultrasonographic examinations were performed by an ECVDI/ACVR (EDI) board certified veterinary radiologist. The hair and skin of each joint was thoroughly soaked with warmed alcohol. A portable ultrasound machine (GE Logiq R7) and a 5–13 MHz Linear Transducer (GE 12 L-RS) was used with focal zone(s), frequency, time gain compensation (TGC) and overall gain optimised by the ultrasonographer. The transducer was held with the marker located medially in a transverse orientation or proximally in the longitudinal plane. Representative cineloop images were recorded and saved for each weanling, facilitating subsequent review.

### Protocol

The order examination was left forelimb, left hindlimb, right hindlimb, and right forelimb. This order allowed optimal sedation during the hindlimb examinations.

### *Carpus* (Fig. 23)

Three transverse oriented imaging planes are obtained.

**Fig. 23:**
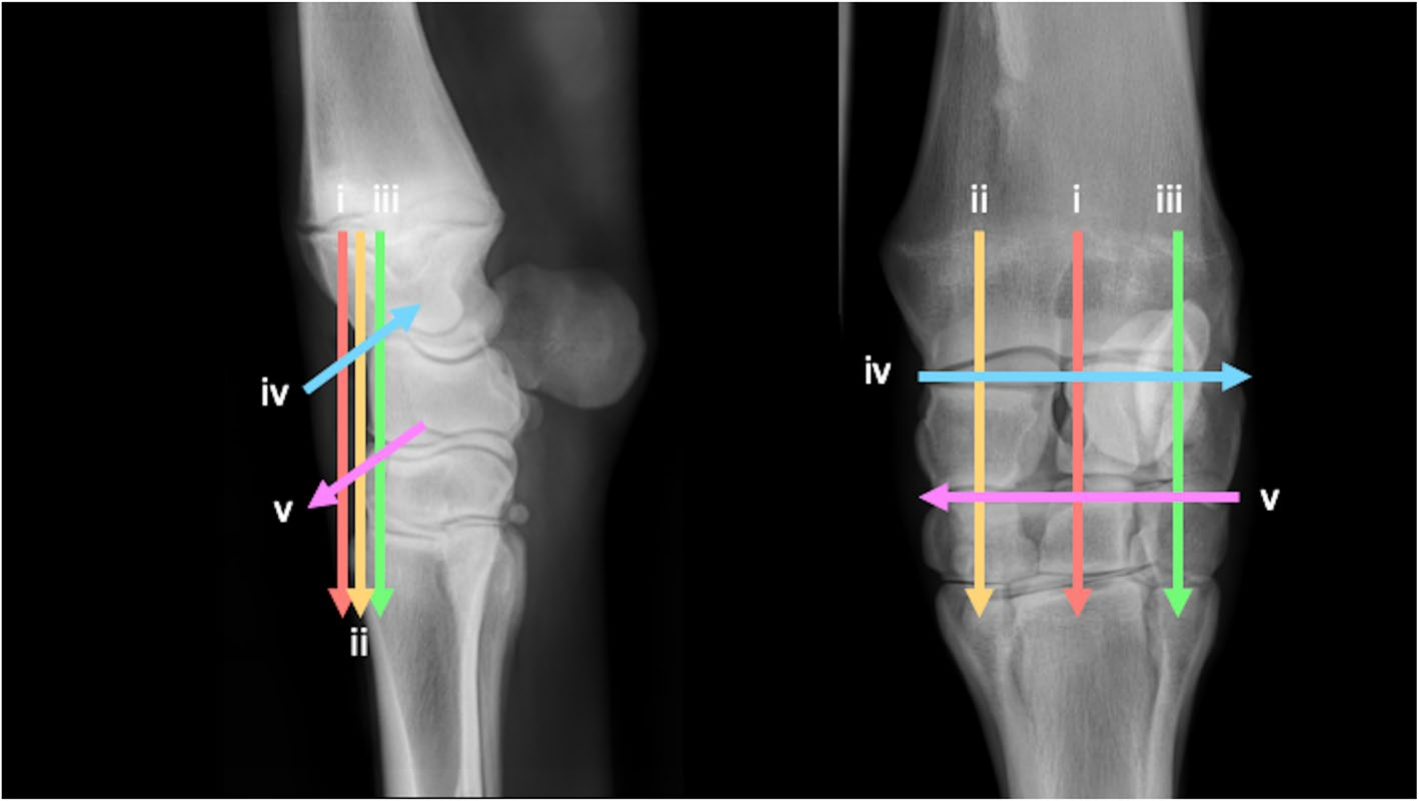
Lateromedial and dorsopalmar radiographic views of the carpus showing the paths of the ultrasound transducer in the examination of the carpus. *Images are acquired in a transverse plane sliding in a proximal to distal direction. Images are acquired in a longitudinal plane sliding in a lateromedial direction. The paths i-v are described in the text*

i. In transverse, identify the extensor carpi radialis tendon (ECRT) dorsal to the distal radial physis. Slide distally, imaging the ABCJ, intermediate carpal bone, MCJ, intermediate facet of C_3_, CMCJ, and proximal MC3.

ii. Return to the ECRT in transverse, at the distal physis of the radius. Slide medially until the tendon is on the lateral aspect of the image. Slide distally imaging the ABCJ, radial carpal bone, MCJ, radial facet of C_3,_ CMCJ and proximal MC2.

iii. Return to the ECRT in transverse orientation, at the distal physis of the radius. Slide lateral to position the tendon on the medial aspect of the image. Slide distally imaging the ulnar carpal bone, fourth carpal bone and proximal MC4.

Two longitudinal oriented imaging planes are obtained.

iv. Rotate 90°into longitudinal plane. Starting at the medial aspect of the ABCJ, slide laterally across the dorsal surface of the ABCJ.

v. From lateral, slide distally to the lateral aspect of the MCJ. Slide medially imaging the dorsal surfaces of the MCJ and CMCJ.

### Metacarpophalangeal joint (Fig. 24)

Three transverse imaging planes are obtained dorsally.

**Fig. 24:**
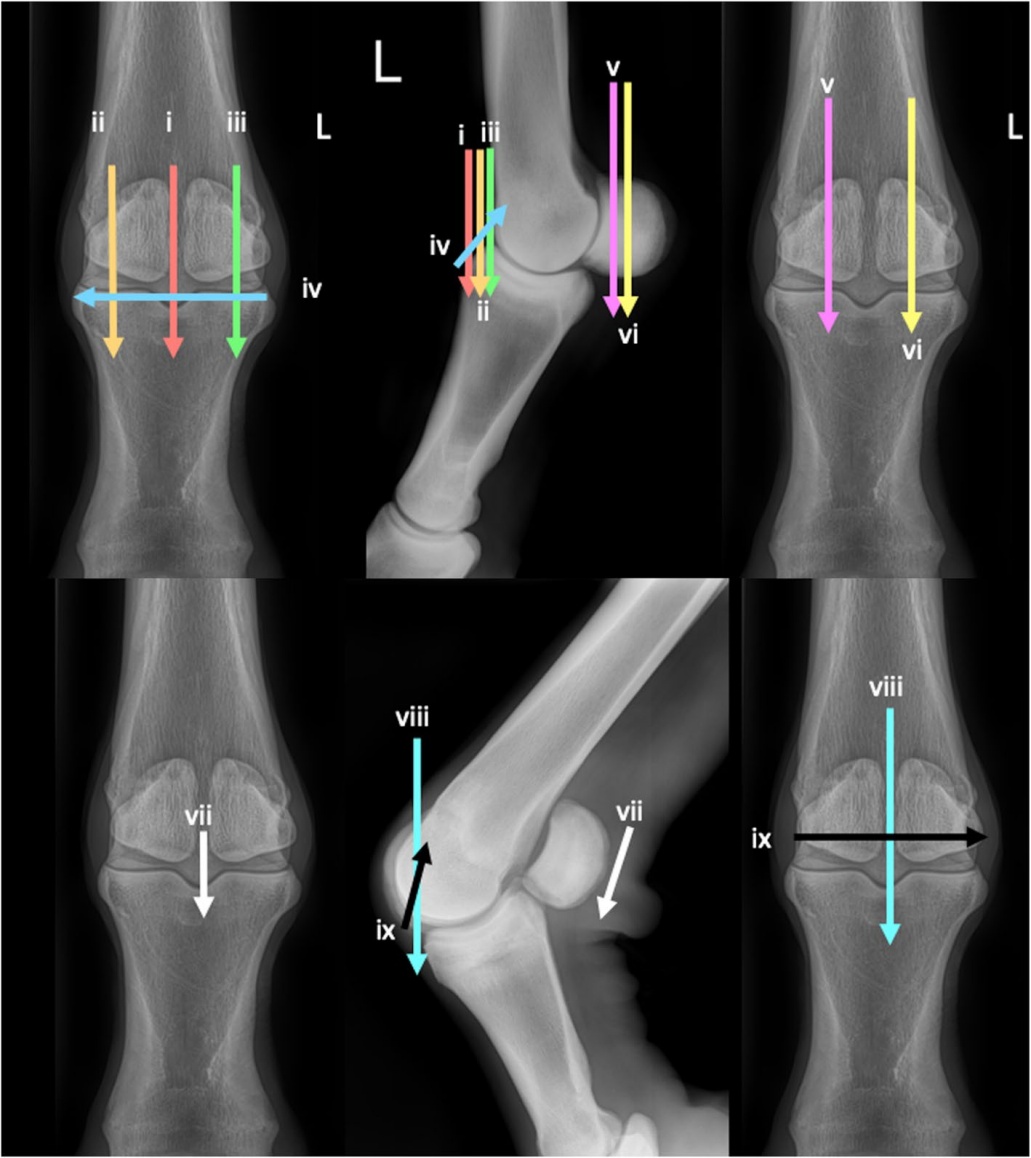
Dorsopalmar, lateromedial and flexed lateromedial views of the metacarpophalangeal joint, showing the paths of the ultrasound transducer in the examination of the metacarpophalangeal joint. *Images are acquired in transverse plane extending in a proximal to distal direction, and in longitudinal plane extending in a lateromedial direction. The paths i-ix are described in the text*

i. Begin in transverse, at the dorsoproximal aspect of the MC_3_ sagittal ridge, at the chondro-osseous margin. Slide distally imaging the sagittal ridge, MCPJ and the P_1_.

ii. Return to midline of the distal MC_3,_ to the level of the chondo-osseous margin. Slide laterally to the lateral aspect of the chondro-osseous margin. From there, slide distally imaging the entire lateral condyle.

iii. Return to the distal MC_3_, to the level of the chondo-osseous margin. Slide medially to the medial aspect of the chondro-osseous margin. From there, slide distally to image the medial condyle.

One longitudinal imaging plane is obtained dorsally.

iv. Return to the distal MC_3_ chondro-osseous margin beginning at the medial aspect, and rotate 90°into longitudinal orientation. From here slide laterally to image the medial and lateral aspects of the MC_3_ condyle, the dorsoproximal margin of the P_1_ and the dorsal margin of the MCPJ.

One longitudinal and one transverse plane are obtained of the branches of the tendon of the third interosseous medius muscle (suspensory ligament) to the palmar eminences of P_1_.

v. In longitudinal, slide distally imaging the entire lateral branch of the tendon of the third interosseous medius muscle (suspensory ligament) to its insertion on the lateral sesamoid bone. Continue sliding distally along the lateral sesamoid to the lateral palmar eminence of P_1_.

vi. Return to the distal aspect of the lateral branch of the tendon of the third interosseous medius muscle (suspensory ligament) and rotate 90° into transverse plane. Slide distally imaging the lateral sesamoid and the lateral palmar eminence of P_1_.

Repeat the process on the medial aspect of the limb.

Two transverse planes and one sagittal plane are obtained with partial limb flexion.

Lift the foot, flexing the MCPJ.

vii. Begin in transverse at the palmar midline of the fetlock, imaging the palmaroabaxial margins of the medial and lateral sesamoids and the Intersesamoidean ligament. Using the intersesamoidean ligament as a window, evaluate the accessible portion of the palmar sagittal ridge of MC3. Sliding distally, image the palmaroproximal aspect of P_1_.

viii. Remaining in flexion, return the transducer, in transverse orientation, to the dorsal mid-line surface of the fetlock imaging the dorsoproximal aspect of the sagittal ridge of MC_3_. Slide from proximal to distal to assess the sagittal ridge and dorsoproximal P_1_.

ix. Return to the dorsomedial aspect of the distal MC_3_ and rotate 90°into longitudinal orientation. Beginning medially, slide dorsally and laterally to image the sagittal ridge of the MC_3_, the dorsoproximal margin of the P_1_ and the dorsal margin of the MCPJ.

### Stifle joints (Fig. 25)

Two transverse planes are imaged of the femoral TRs and condyles.

**Fig. 25:**
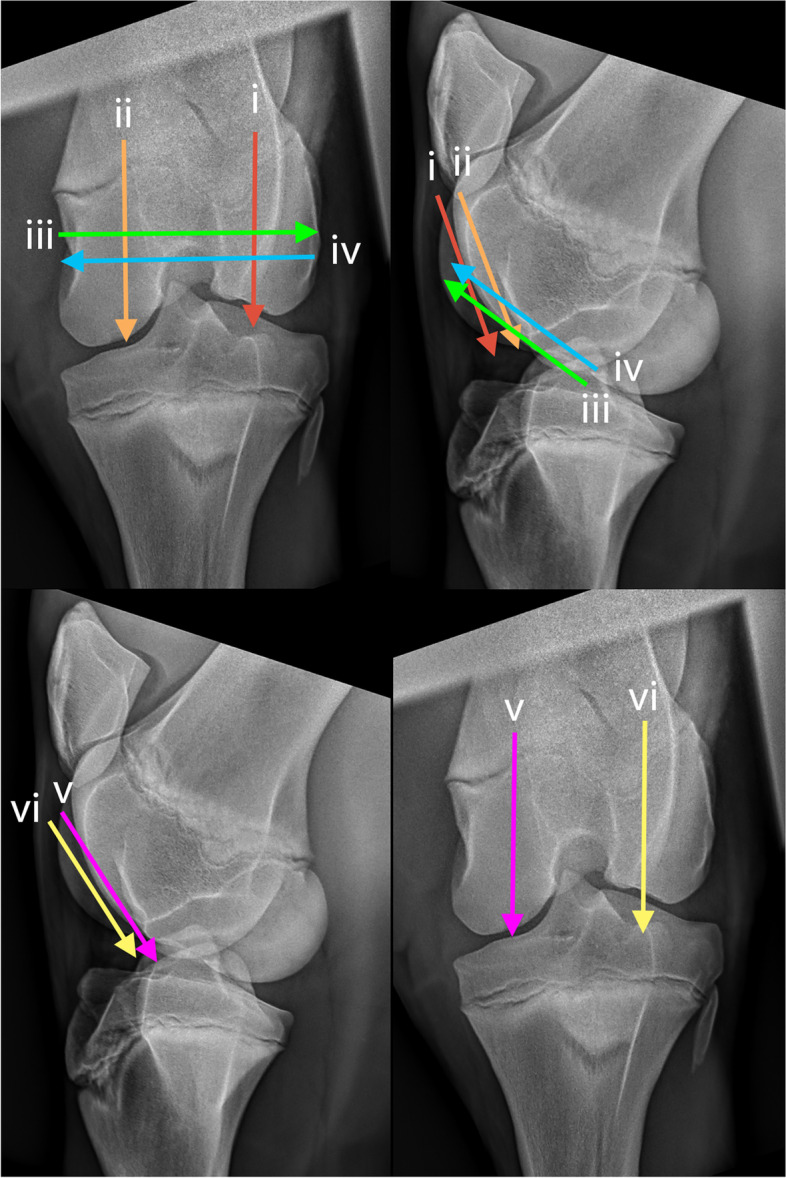
Caudocranial and caudolateral-craniomedial oblique views of the stifle, showing the paths of the ultrasound transducer in the examination of the stifle joint. *Images are acquired in transverse plane extending in a proximal to distal direction, and in longitudinal plane extending in a lateromedial direction. The paths i-vi are described in the text*

In transverse identify the cranial ridge of the patella at its craniolateral aspect.

i. Slide laterally placing the cranial ridge of the patella to the medial aspect of the image. Slide distally to image the lateral femoral TR.

ii. Return to the patella and slide medially, placing the cranial ridge of the patella to the lateral aspect of the image. Slide distally to image the medial femoral TR.

Two longitudinal planes are imaged.

iii. Rotate into a longitudinal plane. Place the transducer at the medial aspect of the femorotibial joint to identify the medial meniscus. Slide slightly proximal to image the medial femoral condylar margin and the recess of the femorotibial joint. From this point, slide cranially, imaging the medial femoral condyle and TR. If the entire femoral trochlear ridges are not included in the imaging plane, a subsequent examination may be repeated placing the transducer more proximally.

iv. Continue to slide laterally to the extensor fossa at the abaxial aspect of the lateral femoral condylar surface. Slide craniodistally approximately 2 cm and rotate approximately 45° in a cranioproximal-caudodistal orientation, to better identify the lateral meniscus, and adjacent femoral and proximal tibial condyles. Slide cranially and medially imaging the lateral femoral condyle and lateral TR and proximal tibial margins.

One transverse plane is imaged in flexion.

The hindlimb is lifted to flex the stifle. A second person, or a hoof stand may be necessary.

v. In transverse orientation, begin at the distomedial aspect of the femur, at the proximal margin of the medial femoral TR. Slide distally to image the medial femoral TR.

vi. Repeat for the lateral femoral TR. At the distal-most aspect the beam is angled caudoproximally to include as much of the condylar surfaces as possible.

### *Tarsus* (Fig. 26)

Three transverse imaging planes are obtained.

**Fig. 26:**
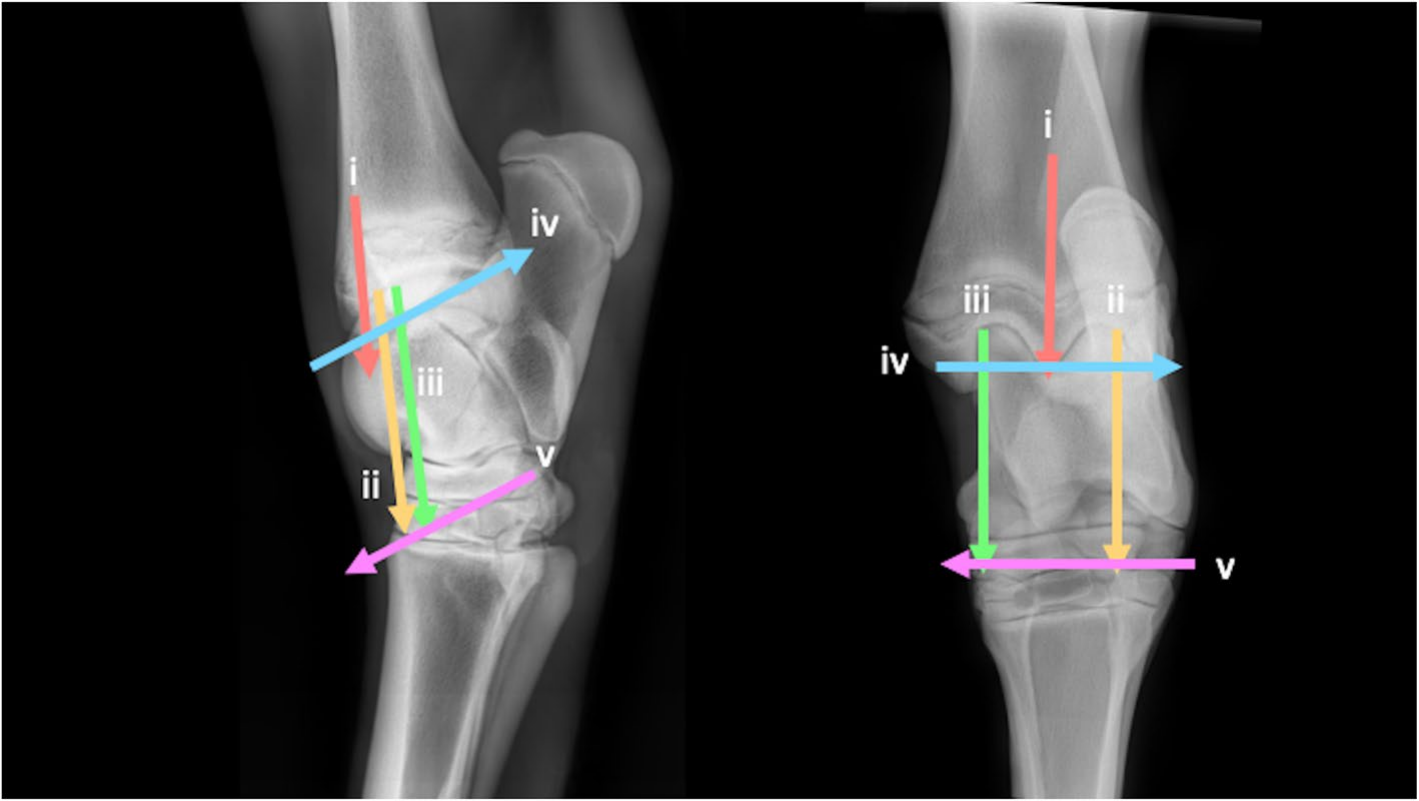
Lateromedial and plantarodorsal views of the tarsus, showing the paths of the ultrasound transducer in the examination of the tarsal joints. *Images are acquired in transverse plane extending in a proximal to distal direction, and in longitudinal plane extending in a lateromedial direction. The paths i-v are described in the text*

i. In transverse, at the craniodistal midline aspect of the tibia, identify the fibularis tertius tendon and slide distally to image the DIRT. The transducer may require proximomedial to distolateral rotation between the TRs of the talus to fully identify the DIRT.

ii. Remaining in transverse, slide laterally to the centre of the lateral TR. Slide distally to assess the lateral talus TR.

iii. Return to the DIRT in transverse plane, sliding medially to centre on the medial talus TR. Slide distally to assess the medial talus TR.

Two longitudinal imaging planes are obtained.

iv. Rotate 90° into longitudinal, slide to the medial aspect of the tarsocrural joint, to the medial malleolus, at the articulation with the medial trochlear ridge of the talus. A slight proximomedial to distolateral transducer rotation may improve surface contact. From here slide laterally imaging the medial TR, DIRT and lateral TR. Mild proximal rocking of the transducer helps to better assess the margins of the DIRT.

v. Slide plantarodistally to the plantar tubercle of the fourth tarsal bone. From this point, slide medially imaging the proximal intertarsal, distal intertarsal and tarsometatarsal joints.

### Metatarsophalangeal joint

As the metacarpophalangeal joint.

### Screening protocol pilot

Two equine veterinary practitioners were recruited. The practitioners performed the examination protocol twice; immediately after viewing a complete protocol demonstration and again six weeks later. Examination duration and images were recorded.

The initial examination did not include the flexed stifle, subsequently included in the second examination. Each observer completed a questionnaire (see supplemental information) relating to their previous ultrasound experience and the difficulty of the examination.

## Supplementary Information

**Additional file 1.**

## Data Availability

All data generated or analysed during this study are included in this published article.

## Abbreviations

ECRT: Extensor carpi radialis tendon
ABCJ: Antebrachiocarpal joint
MCJ: Middle carpal joint
C3: Third carpal bone
CMCJ: Carpometacarpal joint
MCPJ: Metacarpophalangeal joint
P1: Proximal phalanx
MC3: Third metacarpal bone
MC2: Second metacarpal bone
MC4: Fourth metacarpal bone
TR: Trochlear ridge
DIRT: Distal intermediate ridge of the tibia
