# Supplementary material for: Ultrasound screening protocol for osteochondrosis at selected predilection sites in thoroughbred yearlings
Source: Ir Vet J. 2022 Apr 27;75:8. doi: 10.1186/s13620-022-00216-7 (PMC9044857; doi:10.1186/s13620-022-00216-7)
Supplement: Supplementary file 1 — Additional file 1. [file 13620_2022_216_MOESM1_ESM.docx]

| **Equine Joint Ultrasound Survey Technique** | **Answer** | **Score** |
| --- | --- | --- |
| **Operator details:** |  |  |
| Name | ____________________________________ | |
| Please indicate the number of years that you have worked as a veterinary practitioner | ____ | years |
| How experienced are you performing ultrasound examinations | ____ | 1 to 5: 1 inexperienced to 5 very experienced |
| How experienced are you performing ultrasound examinations of equine joints | ____ | 1 to 5: 1 inexperienced to 5 very experienced |
|  |  |  |
| **Joint Ultrasound Survey Technique** |  |  |
| How would you grade the level of difficulty for the overall technique | ____ | 1 to 5: 1 very difficult to 5 very easy |
|  |  |  |
| In each of the following joints, indicate the level of difficulty to acquire standardised images | | |
| Carpus | ____ | 1 to 5: 1 very difficult to 5 very easy |
| Metacarpophalangeal joint |  |  |
| Static | ____ | 1 to 5: 1 very difficult to 5 very easy |
| flexion | ____ | 1 to 5: 1 very difficult to 5 very easy |
| Stifle |  |  |
| Static | ____ | 1 to 5: 1 very difficult to 5 very easy |
| flexion | ____ | 1 to 5: 1 very difficult to 5 very easy |
| Tarsus | ____ | 1 to 5: 1 very difficult to 5 very easy |
| Metatarsophalangeal joint |  |  |
| Static | ____ | 1 to 5: 1 very difficult to 5 very easy |
| Flexion | ____ | 1 to 5: 1 very difficult to 5 very easy |
| **Any Comments** |  |  |
|  | | |
|  |  |  |
